# The mir-21 Inhibition Enhanced HUVEC Cellular Viability during Hypoxia-Reoxygenation Injury by Regulating PDCD4

**DOI:** 10.1155/2022/9661940

**Published:** 2022-06-30

**Authors:** Md Sayed Ali Sheikh

**Affiliations:** Department of Internal Medicine, College of Medicine, Jouf University, Sakaka, Saudi Arabia

## Abstract

The purpose of this study was to explore the clinical value of altered plasma mir-21 expression level as a biomarker for the severity of coronary artery disease (CAD) and its molecular impact on HUVEC cellular injuries. Angiographically validated 56 patients with single-vessel CAD disease, 92 patients with double-vessel CAD, 139 complex coronary artery stenosis patients, and 56 healthy individuals (*n* = 343) were enrolled in this study. The expressions of plasma mir-21 were evidently and progressively higher while PDCD4 levels were significantly and steadily lower in single-, dual-, and multivessel occluded CAD patients than in healthy participants (*P* < 0.001). The relative expressions of mir-21 in hypoxia-reoxygenation- (HR-) exposed HUVECs were markedly upregulated, but PDCD4 concentrations were obviously downregulated as compared with normal control cells (*P* < 0.001). Moreover, altered circulatory mir-21 expression levels were able to significantly differentiate single- (AUC 0.893), double- (AUC 0.914), and multivessel stenosis CAD (AUC 0.933) patients from healthy subjects. Besides, the plasma mir-21 expressions in elderly (66-85 years) groups were remarkably higher than those in younger aged (25-45 years) subjects. Caspase-3 and ROS expression levels were remarkably elevated, but cellular viability noticeably declined in HR-induced HUVECs than in normoxic cells (*P* < 0.001). In contrast, mir-21 inhibition markedly reduced caspase-3 activity and ROS concentrations while significantly ameliorating HUVEC cellular viability in HR conditions. PDCD4 expressions in HR-exposed HUVECs were prominently decreased whereas mir-21 inhibition significantly enhanced PDCD4 levels (*P* < 0.001). Upregulated plasma mir-21 can be a valuable clinical biomarker for the detection of the severity of coronary artery stenosis patients. Elevated circulatory mir-21 concentrations have a positive correlation with aging. Inhibitory mir-21 evidently increased HUVEC cellular viability through upregulation of targeting PDCD4 and recommended a newer possible therapeutic molecule for the management of CAD patients.

## 1. Introduction

Atherosclerotic coronary artery disease (CAD) is the principal reason for unexpected death among adults in all ethnic groups worldwide. Due to the high incidence of unhealthy lifestyle, often poorly treated major atherosclerotic risk factors, and substantial impact of genes, by the next 10 years, approximately 12% CAD prevalence rates will be increased [1]. Early diagnosis with advanced management and also adequate control of CAD risk factors will greatly decline the CAD-associated death incidence and significantly reduced hospital admission rates and economic burden. Currently, invasive coronary angiogram (CAG) and percutaneous coronary intervention (PCI) are widely reliable clinically used techniques for the evaluation and management of ischemic coronary heart disease, respectively. However, invasive CAG has some surgical and technical complications, and it is not suitable for all the patients, especially older chronic kidney disease patients. Moreover, PCI management is also not suitable for multivessel occluded CAD patients, even in complicated left main artery (LM) CAD lesion subjects. Therefore, noninvasive universal early diagnostic biomarkers and newer therapeutic drugs are clinically demanded by cardiologists.

MicroRNAs (miRNAs, mirs) are highly conserved, endogenous, single-stranded, small noncoding RNAs of about 18-24 nucleotides in length that regulate gene expression via either inhibiting the translation of messenger RNAs(mRNAs) or degradation of mRNAs. In recent years, several research studies have confirmed that various miRNAs are directly regulated in the entire cardiovascular physiology and also exhibit an essential role in the many cardiovascular disease processes including CAD. Coronary artery disease usually occurred due to chronic lipid-induced atherosclerotic inflammation in the arterial wall, and several atherosclerotic-related miRNAs (mir-342-5p, mir-217, mir-126, mir-155, and mir-92a-3p) are critically involved for the formation and subsequent rupture of the coronary atherosclerotic plaque [2–4].

It has been reported that dysregulated mir-21 was essentially implicated in endothelial dysfunction, progression of early to advance atherosclerotic plaque, formation of critical coronary stenosis, and thrombus leading to acute coronary syndrome through activation of different inflammatory cells, cytokines, and signaling pathways including macrophages, tumor necrosis factor-*α* (TNF-*α*), interleukin- (IL-) 17A, IL-1*β*, IL-6, phosphatase and tensin homolog (PTEN), PI3K/Akt MAPK, vascular endothelial growth factor, and TLR4/NF-*κ*B [5–9].

Programmed cell death 4 (PDCD4) protein was initially discovered as a novel upregulated gene during apoptosis, and it acts as a regulator of gene expression by the influencing translation and transcription process. It has been recently demonstrated that PDCD4 is widely expressed in myocardial cells, vascular smooth muscle cells, and endothelial cells, and PDCD4 directly regulated various inflammatory pathways including adipose inflammation, endothelial dysfunction, foam cell formation, and atherosclerosis through altering differing cytokine expressions such as interleukin-10 (IL-10), IL-17, IL-6, IL-1*β*, IL-8, nuclear factor-kappa B (NF-*κ*B), tumor necrosis factor-alpha (TNF-*α*), and NOTCH1. Moreover, emerging evidence has shown that PDCD4 is directly involved in coronary atherosclerotic plaque formation, and its expression levels were significantly attenuated in atherosclerotic coronary artery disease [5, 10–12].

Moreover, our previous study and also some other research studies have demonstrated that circulatory mir-21 expression patterns were evidently altered in significant or insignificant coronary artery stenosis, unstable angina, and acute coronary syndrome with a strong relationship with aging [7, 8, 13, 14]. It has been explored that hypoxia-reoxygenation- (HR-) induced human umbilical vein endothelial cell (HUVEC) damage is noticeably prevented by inhibition of miR-21 expression through regulating SMAD7. Shao et al. revealed that inhibitor mir-21 prominently reduced the atherosclerotic lesion area by the RAGE/NADPH signaling pathway via regulating ADAM10 expression in the mouse model, suggesting a novel therapeutic target for the treatment of atherosclerotic disease. Another study showed that inhibitory mir-21 protects cardiomyocyte apoptosis and attenuated the infarct size in both cellular and mouse models of AMI by regulating PDCD4 expression, providing a newer therapeutic strategy for AMI treatment [15–17].

However, the association between mir-21 and PDCD4 in the severity of coronary artery lesions and HR-induced HUVEC cellular damage remains to be elucidated. The aim of the present research examined the impact of deregulated circulatory mir-21 concentrations in the categorized coronary artery stenosis patients and disclosed its molecular protective mechanisms against H/R-induced HUVEC cellular injury.

## 2. Materials and Methods

### 2.1. Study Subjects

Angiographically proven 56 single-coronary artery stenosis, 92 dual-vessel stenosis, 139 multivessel blocked CAD patients, and 56 healthy volunteers were collected between January 2016 and May 2017 from the 1^st^ Affiliated Xiangya Hospital, Central South University. CAD was considered if coronary artery blockade was observed at ≥50% through the invasive coronary angiogram by two interventional cardiologists. Single-vessel disease is defined when one major coronary (LAD, LCX, and RCA) artery occluded, dual-vessel disease is considered when two major coronary arteries or individually blocked left main (LM) artery occluded, and multivessel lesion CAD patients were categorized when two or more than two major coronary arteries including their branches were occluded. Acute or chronic heart failure, acute coronary syndrome, stroke, implanted pacemaker or ICD, revascularization by coronary stent or open-heart surgery (CABG), cardiomyopathy, and pulmonary hypertension subjects were excluded from this study. Individuals with no history of cardiovascular diseases, chronic renal insufficiency, chronic liver, or other multisystem inflammatory disorders or malignancy were recruited as healthy participants. Prior to this study, from all the human study subjects, written informed consent was obtained, and the study was conducted by following the human research protocols in the Declaration of Helsinki. Human and cellular investigations of this study were reviewed and approved by the Ethics Committee of Xiangya Hospital, Central South University. 5 mL peripheral venous blood samples was obtained by percutaneous cubital venipuncture in EDTA-coated tubes from all the participants.

### 2.2. Cell Culture, Transfection, and Dual-Luciferase Reporter Gene Assay of HUVEC

The primary human umbilical vein endothelial cell line (HUVEC) was purchased from the Chinese Academy of Sciences (Shanghai, China) and cultured in 12-well plates at a density of 5 × 10^5^ cells/well by using Dulbecco's Modified Eagle's Medium (DMEM) supplemented with 20% fetal bovine serum (FBS) in a cell incubator with maintained normoxic conditions at 37° C, 5% CO_2_, and 95% air. The hypoxia-reoxygenation (HR) model was established by harvesting HUVECs in an anaerobic modular incubator containing 95% N_2_, 5% CO_2_, and 1% O_2_, using serum and glucose-free DMEM for 12 hours; afterward, cells were reoxygenated for 6 hours in a normoxic incubator. HUVECs were transfected with has-inhibitory-miR-21 (100 nmol/L) for inhibition, and has-inhibitory-negative control- (NC-) mir-21 (100 nmol/L) (RiboBio, Guangzhou, China) acts as an inner control by using the Lipofectamine 2000 transfection agent (Invitrogen, USA); more details are shown in our prior studies [14, 18, 19].

The luciferase reporter gene analysis technique was used to confirm the target gene of mir-21. The complementary sequence of 3′-UTR of PDCD4 was predicted with mir-21 by TargetScan (http://www.targetscan.org/). Lipofectamine 2000 reagents and has-inhibitory and negative controls-mir-21 were used for the transfection of HUVECs, and dual-luciferase gene analysis chemicals (Beyotime, Jiangsu, China) were applied for the determination of HUVEC luciferase activities normalized by Renilla and fluorescent activity was used for the measurement of their ratio.

### 2.3. Intracellular ROS, Caspase-3 Activity, and Cellular Viability Assays

Healthy, hypoxia-reoxygenation, and transfected HUVEC cellular ROS concentrations were demonstrated by adding the 5 *μ*M dichlorodihydrofluorescein diacetate (DCFH-DA) solution into each 96-well black plate after proper growth of the cells and inoculated for 30 minutes at 37°C. Afterward, two times cells were cleaned by PBS, and finally, ROS values were measured using an ultraviolet SpectraMax microplate reader (Molecular Devices, Sunnyvale, CA) at the 490 nm wavelength.

Caspase-3 activities were performed by using the caspase-3 activity colorimetric kit according to the manufacturer's guidelines (Beyotime, Shanghai, China). Moreover, 100 microliters of caspase-3 substrate was added to each normal and treated 96-well plate HUVECs. All the samples were incubated at 37°C for 2 hours, and caspase-3 expression levels were evaluated through a microplate SpectraMax absorbance reader (Molecular Devices, Sunnyvale, CA) at the wavelength of 405 nm.

Cellular viability and proliferation were assessed by using CCK-8 kits (Beyotime, Shanghai, China) by following the company's protocols. The HUVECs were cultivated into 96-well plates; after proper treatment, 10 microliters of the CCK-8 reagent was mixed and inoculated at 37°C for 2 hours; then, the normal, HR, and HR plus treated HUVECs were washed twice by PBS; subsequently, cellular viability was detected at the wavelength of 450 nm by using a SpectraMax (Molecular Devices, Sunnyvale, CA) absorbance reader microplate.

### 2.4. Detection of miRNA and mRNA Levels

Pure RNAs were extracted with TRIzol solution from circulating human plasma, normal, HR, and HR with transfected HUVECs (Invitrogen, CA, USA). The hsa-mir-21, inhibitory and negative control- (NC-) mir-21, and internal reference miR-156a primers were designed by Guangzhou RiboBio (China). The primers of PDCD4 and endogenous reference *β*-actin were obtained from Shanghai (China) Biotech. Target protein primer sequences are presented in Table 1. Moreover, a total of 4 *μ*L of pure RNA (OD: 1.8-2.2, nucleic acid concentration: 50-500 *μ*g) was reverse-transcribed (RT) to cDNA at 42°C for 30 minutes using the Bulge-Loop miRNA-specific reverse transcription primers (RiboBio, Guangzhou, China) as per instructions of the manufacturer through the RT-PCR system (Bio-Rad, USA). Subsequently, 2 *μ*L of cDNA was used as the template in a real-time quantitative PCR reaction by the following thermal parameters: an initial incubation at 95°C for 15 s, followed by 40 cycles of denaturation at 95°C for 5 s and annealing and extension at 60°C for 31 s. The relative expressions of mir-21 concentrations and PDCD4 levels were analyzed with Master Mix SYBR Green qRT-PCR reagents (Takara Bio, Dalian, China) through a 7300 Real-Time PCR System (Applied Biosystems, Thermo Fisher Scientific, USA.). Moreover, the RNA isolation and qRT-PCR procedures for miRNA and mRNA expressions were more discussed in our prior studies [18, 19].

### 2.5. Statistical Data Analysis

All the data in this study were expressed as mean ± standard deviation (SD) or SEM, and at least three independent experiments were performed for each data and analyzed through SPSS 22.0 and GraphPad Prism 8.0. To analyze the continuous variation within two groups, the 2-tailed Student *t*-test and the nonparametric Mann-Whitney test were performed, and for categorical variations, Fischer and the chi-square (*χ*^2^) tests were implemented, and the differences between more than two groups were measured by one-way analysis of variance (ANOVA). The sensitivity and specificity of mir-21 as a biochemical marker were examined by the Receiver Operating Characteristic (ROC) curve analysis method. A value of *P* < 0.05 was considered to indicate a statistically significant difference.

## 3. Results

### 3.1. Baseline Clinical and Laboratory Information of All the Study Subjects

There were 56 patients (35 males, 21 females) with single-vessel blocked CAD, 92 (52 males, 40 females) patients with dual-vessel lesion CAD, 139 patients (76 males, 63 females) with multivessel occluded CAD, and 56 (28 males, 28 females) well-coordinated gender- and age-matched healthy individuals who were enrolled in this study, and their (*n* = 343) baseline clinical features are presented in Table 2. Lipid profiles (LDL, HDL, TC, and TG), serum creatinine, and C-reactive protein values were reasonably higher in multivessel CAD patients than in healthy subjects. However, other clinical information among healthy, single, dual, and complex lesion CAD subjects was not statistically significant (Table 2).

### 3.2. Expression of mir-21 and PDCD4 in Plasma and HUVECs

**Figure 1:**
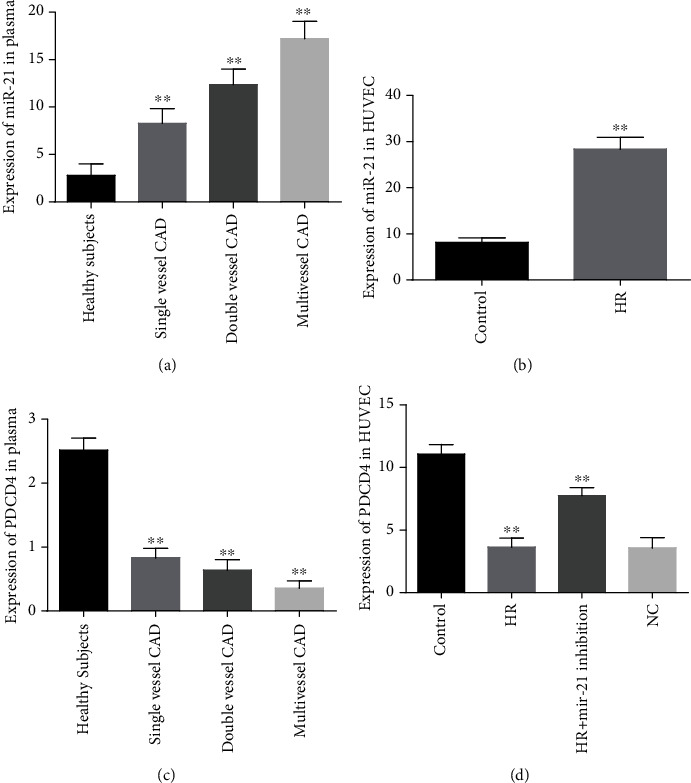
The expression patterns of mir-21 and PDCD4. (a) Circulating mir-21 concentrations in healthy participants versus single-, double-, and multivessel lesion CAD subjects. (b) Expression levels of mir-21 among hypoxic reoxygenation (HR) and normal control HUVECs. (c) The PDCD4 expression patterns between healthy volunteers and disease groups. (d) Comparison of PDCD4 expressions in HR-exposed and normoxic-cultured HUVECs. ^∗∗^*P* < 0.001.

The expressions of circulating plasma mir-21 levels were progressively elevated in single-, dual-, and multivessel occluded stable CAD patients as compared with healthy individuals. The highest levels of mir-21 were found in complex-vessel lesion CAD groups compared with double- and single-vessel CAD subjects, and they were statistically significant (*P* < 0.001) (Figure 1(a)). The relative mir-21 expressions in hypoxic reoxygenation- (HR-) exposed HUVECs were significantly upregulated by 3.4-fold (29.02 ± 1.4) compared to those in normal atmospheric-cultured healthy cells (8.44 ± 0.63) (*P* < 0.001) (Figure 1(b)). The expressions of circulating PDCD4 were gradually reduced in single-vessel stenosis and double- and multivessel occluded CAD groups as compared with healthy subjects. The lowest concentrations of PDCD4 were recorded in multivessel CAD groups as compared with those with double- and single-vessel CAD groups. Moreover, PDCD4 expressions within the different CAD disease groups were also evidently significant (*P* < 0.001) (Figure 1(c)). In HR-induced HUVECs, PDCD4 levels were markedly downregulated by 3.2-fold (11.23 ± 0.51) compared to those in control cells (3.47 ± 0.76) (*P* < 0.001) (Figure 1(d)).

### 3.3. Clinical Significance of Plasma mir-21 in the Grading of CAD Patients

**Figure 2:**
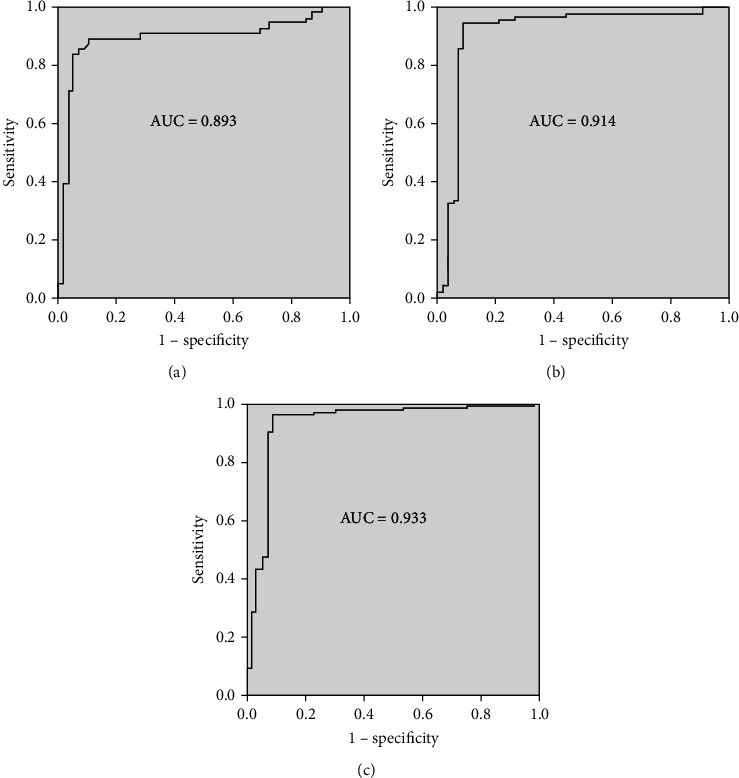
ROC curve analysis was used for categorizing the severity of CAD patients: (a) healthy controls and single-vessel lesion CAD (AUC 0.893); (b) dual-vessel stenosis CAD with healthy subjects (AUC 0.914); (c) healthy volunteers and multivessel blocked CAD patients (AUC 0.933).

The clinical role of circulating mir-21 to detect the severity of CAD patients was assessed through the ROC method. In single-vessel occluded CAD subjects, plasma mir-21 expressions were able to strongly discriminate from those in healthy subjects with a prominent area under the curve (AUC) of 0.893. In dual-vessel lesion CAD patients, circulating mir-21 levels were evidently separated from those in healthy participants with a significant AUC of 0.914. The plasma mir-21 concentrations in multivessel blocked CAD patients were noticeably distinguished from those in control subjects (AUC of 0.933) with high specificity and sensitivity levels. The AUC curves of plasma mir-21 were also distinctly significant and had a strong predictive power to differentiate between single-, double-, and multivessel blocked CAD patients (Figure 2). These findings suggested that upregulated plasma mir-21 has a significant clinical diagnostic role to categorize the severity of CAD patients.

### 3.4. Expression Profile of mir-21 in HUVECs and Luciferase Assay

**Figure 3:**
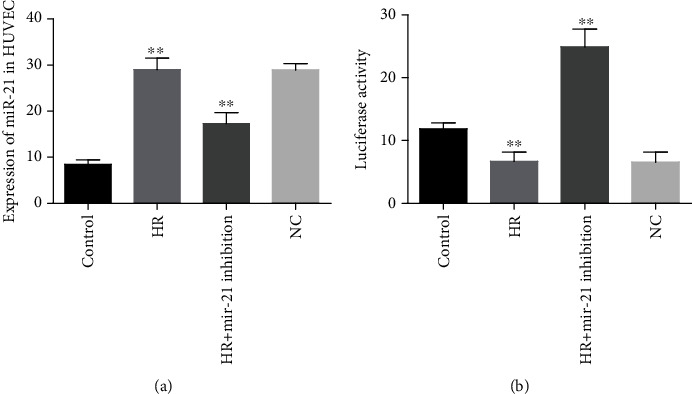
Expression patterns of mir-21 in HUVECs and luciferase analysis to determine the target: (a) the mir-21 expression levels in healthy and HR groups; (b) reporter gene assay luciferase expression levels in normoxic, HR, HR with transfected mir-21, and NC groups in HUVECs standardized by Renilla reniformis. ^∗∗^*P* < 0.001.

The relative expressions of mir-21 were markedly upregulated in HR-exposed HUVECs as compared with healthy control cells (*P* < 0.001). In contrast, mir-21 levels were noticeably downregulated by 1.7-fold (17.32 ± 2.3) in HR-injured HUVECs transfected with inhibitory mir-21 as compared with HR-incubated HUVECs (29.02 ± 2.4), but expression profiles within negative control (NC) and HR-incubated groups were nonsignificant (Figure 3(a)). Furthermore, the current study also demonstrated that luciferase activity levels were evidently decreased in HR-incubated HUVECs by 1.8-fold (6.68 ± 1.4) compared to normoxic cells (11.84 ± 0.9), but no significant differences were observed between NC and HR groups. The inhibitory mir-21 strongly controlled luciferase activities in HR-injured HUVECs (Figure 3(b)).

### 3.5. The Inhibitory mir-21 Regulated PDCD4 Expression and Augmented HUVEC Survival

**Figure 4:**
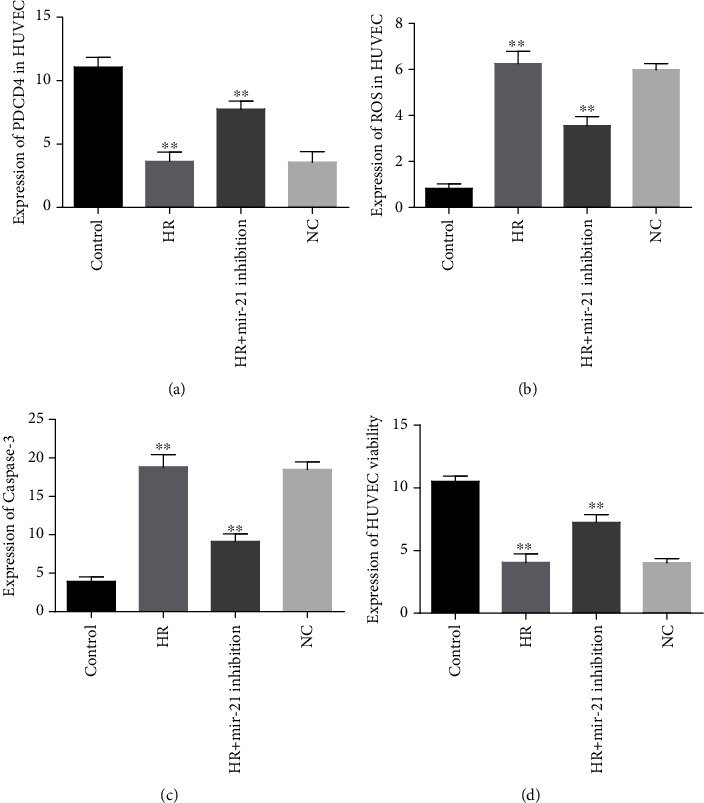
Effects of mir-21 inhibition on HUVECs: (a) the relative expression levels of PDCD4 in normoxic and HR cells; (b) intracellular ROS generations in normal HUVEC versus HR-injured cells; (c) caspase-3 expression between normal culture and HR-incubated cells; (d) cellular viability in HR and normal, HR and HR transfected with mir-21 inhibition, and HR and NC groups in HUVECs. ^∗∗^*P* < 0.001.

The relative expressions of PDCD4 levels were prominently downregulated in HR-injured HUVECs whereas they were remarkably upregulated as close to controls upon treatment with inhibitory mir-21. No obvious effects were found between NC and hypoxic-reoxygenated- (HR-) exposed HUVECs, suggesting that PDCD4 expressions were strongly regulated by inhibitory mir-21 in normal and HR-damaged HUVECs (Figure 4(a)).

In HR-exposed HUVECs, the levels of intracellular ROS were much higher than normally cultured cells while amazingly reduced by treated HR-damaged HUVECs with inhibitory mir-21. Among HR and NC groups, no significant effects were revealed on ROS expressions, indicating that inhibitory mir-21 has a major role to prevent oxidative stress-induced HUVEC cellular injures under HR situations (Figure 4(b)). Caspase-3 activities were markedly elevated in HR-exposed HUVECs than in normal cells. On the contrary, HR-induced HUVECs transfected with inhibitory mir-21 caspase-3 expressions were remarkably decreased, but the activities of caspase-3 expressions were insignificantly expressed between NC and HR, acknowledging that mir-21 inhibition is essentially involved to protect against HUVEC cellular damage in HR conditions (Figure 4(c)). The HUVEC cellular viability was significantly decreased in HR-induced groups compared to normal healthy cells. On the other hand, HR-exposed HUVECs treated with mir-21 inhibition remarkably increased cellular viability while no obvious changes were found among NC and HR groups; these findings strongly recommended that inhibitory mir-21 plays a fundamental role in cellular protection against injuries in the HR environment (Figure 4(d)).

### 3.6. Plasma mir-21 Expression Association with Aging and Gender

**Figure 5:**
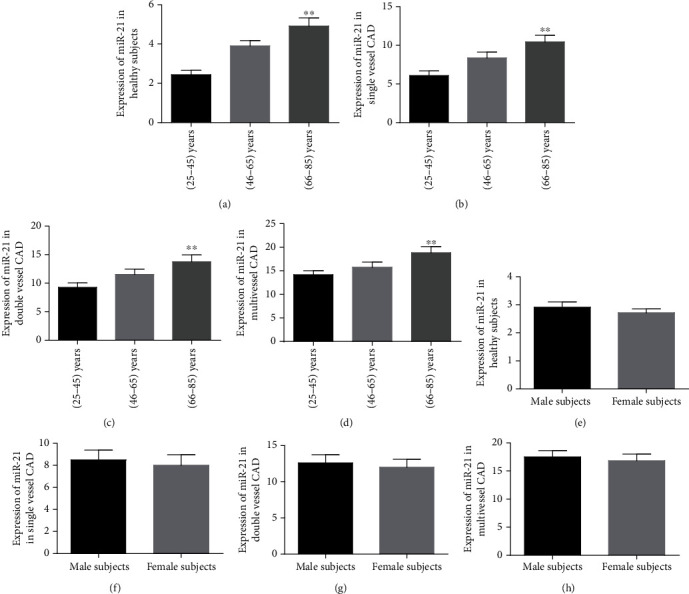
The relative expression of circulatory mir-21 in different aged subjects and both male and female genders: (a) the expression patterns of plasma mir-21 among healthy groups; (b) plasma mir-21 concentrations in single-vessel CAD subjects; (c) the expression of mir-21 levels in double-vessel CAD patients; (d) multivessel CAD patients' mir-21 levels; (e) healthy male and female groups' mir-21 expressions; (f) the levels of plasma mir-21 in male versus female single-vessel CAD groups; (g) the concentrations of circulatory mir-21 among male and female double-vessel stenosis CAD subjects; (h) the mir-21 levels between male and female multivessel blocked CAD patients.

In elderly healthy subjects (66-85 years), circulatory plasma mir-21 concentrations were relatively higher than those in younger healthy participants (25-33 years) (*P* < 0.001) (Figure 5(a)). In single-, double-, and complex-vessel occluded CAD geriatric aged (66-85 years) people, the mir-21 expressions were significantly increased as compared with those in younger aged (25-33 years) people (*P* < 0.001) (Figures 5(b)–5(d)). However, in both genders among healthy and single-, dual-, and multivessel stenosis CAD groups, plasma mir-21 levels were nonsignificant (*P* > 0.05) (Figures 5(e)–5(h)). These findings indicated that upregulated mir-21 expressions have been positively and strongly correlated with aging.

## 4. Discussion

Promoting a healthy lifestyle and early evaluation with immediate proper treatment can significantly decline CAD death incidence. Endothelial dysfunction and various inflammatory mediators are the major molecular insights to develop atherosclerotic heart disease. Over the past two decades, accumulating evidence has elucidated that microRNA plays a vital role in regulating endothelial injury, atherosclerosis, and cellular apoptosis.

This study explored the clinical significance of mir-21 in the severity of atherosclerotic CAD patients, and it is linked with PDCD4 as well as its preventing role during HR-exposed HUVEC cellular injury. The present research study found that mir-21 expressions were obviously and steadily upregulated in the plasma of single-, double-, and complex-vessel lesion CAD patients compared to healthy control subjects. The plasma expressions of PDCD4 were progressively decreased in single-, dual-, and multivessel stenosis CAD participants compared with normal individuals. Significant or insignificant coronary stenosis subject's mir-21 concentrations were obviously higher identified by Ziba et al.'s research group [13]. Our previous stable CAD study subjects demonstrated significantly upregulated circulatory mir-21 concentrations compared to healthy subjects [14]. Moreover, the PDCD4 expression levels in the AMI rat model were evidently lower compared with those in the control groups reported by Zhou et al. [20].

The clinical significance of plasma mir-21 in the prediction of severity of CAD was evaluated by ROC curve analysis. The single-, dual-, and multivessel CAD stenosis patients were able to be significantly differentiated (AUC of 0.893, 0.914, and 0.933, respectively) from the healthy control participants, and their differences within the subgroups were statistically highly significant. These findings suggested that upregulated circulatory mir-21 can be used as a noninvasive diagnostic clinical potential marker for the detection of stenosis levels in CAD subjects, and also, similar results are reported by some clinical experiments [14, 21, 22].

The present study reported that lipid profiles and serum creatine values in multivessel CAD patients were significantly elevated compared to those in healthy controls, but difference within groups were insignificant. The C-reactive protein concentrations in single-, double-, and complex-vessel lesion CAD subjects were remarkably higher than those in healthy individuals. Our previous study also demonstrated that higher CRP levels have a strong association with stable and unstable angina patients that required further molecular studies to discover their undeveloped signaling pathways [14, 19].

This study also revealed that mir-21 expressions were evidently increased in geriatric subjects compared to younger people, suggesting a significant link with the aging process. Wang et al. and also our prior study have identified in elderly subjects remarkably elevated mir-21 and association with the cardiorenal syndrome and aging [14, 23].

It has been established that PDCD4 is crucial in regulating oxidized low-density lipoprotein (ox-LDL) metabolism, foam cell formation, coronary atherosclerosis, stress, and ischemia/reperfusion-induced cellular injuries [10–12, 24]. From our luciferase analysis experiment results, from the TargetScan database, and also from other studies, it was confirmed that PDCD4 is the direct target protein of miR-21 [20, 24, 25]. The present study detected expressions of mir-21 in HR-exposed HUVECs which were highly upregulated as compared with the control group, but there were no significant effects noted between NC and HR groups. Chang et al. also moderately supported this study [15]. Moreover, the current study demonstrated that PDCD4 expressions were evidently decreased in HR-incubated HUVECs while they were markedly elevated after transfecting with mir-21 inhibition. The intracellular production of ROS levels in HR-induced HUVECs was markedly upregulated but significantly dropped back near to normal levels after being treated with mir-21 inhibition. Caspase-3 activities in HR-cultured HUVECs were enormously elevated but noticeably reduced after transfecting with mir-21 inhibition. Zhou et al. revealed mir-21-regulated cardiomyocyte apoptosis via targeting PDCD4 which relatively supported this study [20]. Besides, the present research established that cellular viability and proliferation markedly declined in HR-induced HUVECs compared with normally cultured cells but significantly improved cellular viability after being treated with mir-21 inhibition. All these results confirmed that mir-21 inhibition can protect against HR-induced HUVEC cellular injuries and suggested a new potential therapeutic target for CAD patients.

However, the molecular relationship between mir-21 with lipid profiles and C-reactive protein was not investigated in this study and needs further studies. Additionally, this is a tertiary-level comparatively small sample size single hospital-based one ethnic population study. Therefore, different ethnic multiregional larger population-based clinical cohort experiments will be conducted in the future for the rationality of the clinical importance of circulatory miR-21 as an ideal useful early noninvasive diagnostic tool and possible therapeutic agent for the management of ischemic heart disease.

## 5. Conclusion

>The increased plasma mir-21 expression level could be a novel biomarker for the severity of coronary artery stenosis and correlation with aging, and mir-21 inhibition prevents HUVEC cellular injuries under hypoxia-reoxygenation insults via targeting PDCD4, suggesting a potential target for treating atherosclerotic CAD patients.

## Figures and Tables

**Table 1:** Primer sequences of target proteins and their relationship with mir-21 and PDCD4.

Target genes	Forward sequences (5′ to 3′)	Reverse sequences (5′ to 3′)
Hsa-miR-21	GTGCAGGGTCCGAGGT	GCCGCTAGCTTATCAGACTGATGT
Hsa-miR-156	AGGCGCCTGACAGAAGAGAGT	GTGCAGGGTCCGAGGT
Hsa-PDCD4	AGGCCGAGGTGGGCGGATCACTTGA	GCCACCATGCCTGGCTACT
Hsa-*β*-actin	TGACGTGGACATCCGCAAAG	CTGGAAGGTGGACAGCGAGG

(i) Position 242-249 of PDCD4: 3′-UTR-AAGUGGAAUAUUCUAAUAAGCUA. (ii) Hsa-miR-21: AGUUGUAGUCAGACUAUUCGAU. Abbreviation: Hsa: human.

**Table 2:** Baseline features of the study participants.

Parameters	Healthy participants (*n* = 56)	Patients with single-vessel CAD (*n* = 56)	Patients with double-vessel CAD (*n* = 92)	Patients with multivessel CAD lesion (*n* = 139)	*P*_1_	*P*_2_	*P*_3_
Age (years)	57.5 ± 9.3	58.6 ± 11.7	60.4 ± 11.7	62.5 ± 12.9	0.539	0.217	0.184
Male/female	28/28	35/21	52/40	76/63	0.492	0.113	0.086
Systolic BP (mmHg)	122.3 ± 7.8	130.8 ± 10.7	131.9 ± 12.8	133.8 ± 13.5	0.089	0.326	0.251
Diastolic BP (mmHg)	75.8 ± 6.4	80.2 ± 7.9	81.6 ± 8.7	82.8 ± 8.5	0.091	0.144	0.163
Heart rate (bpm)	75.3 ± 8.6	78.7 ± 6.9	80.4 ± 9.2	81.5 ± 10.7	0.257	0.389	0.446
Body mass index (kg/m^2^)	22.5 ± 5.8	23.7 ± 8.6	23.9 ± 7.4	24.1 ± 6.2	0.428	0.507	0.453
Current tobacco smoking	52% (29)	56% (31)	60% (55)	58% (81)	0.642	0.715	0.783
Lipid disorders	—	86% (48)	88% (81)	92% (128)	—	—	—
Diabetes mellitus	—	28% (16)	32% (29)	34% (47)	—	—	—
Essential hypertension	—	57% (32)	66% (61)	68% (95)	—	—	—
LDL-C (mmol/L)	2.32 ± 0.4	2.65 ± 1.2	3.2 ± 1.7	3.98 ± 1.3	0.126	0.079	<0.001
TCHO (mmol/L)	4.7 ± 0.3	5.12 ± 0.6	5.53 ± 0.4	5.76 ± 0.9	0.078	0.092	<0.001
HDL-C (mmol/L)	1.6 ± 0.9	1.03 ± 0.7	0.89 ± 0.4	0.71 ± 0.8	0.099	0.086	<0.001
TG (mmol/L)	1.29 ± 0.4	1.58 ± 0.6	1.71 ± 0.5	2.35 ± 0.9	0.084	0.065	<0.001
Serum creatinine (mg/dL)	0.78 ± 1.29	1.31 ± 1.2	1.34 ± 1.4	1.47 ± 1.5	0.122	0.096	<0.001
Left ventricular ejection fraction	59.4 ± 11.3	53.7 ± 8.6	50.9 ± 10.7	50.3 ± 11.4	0.125	0.096	0.087
C-reactive protein (mg/L)	3.7 ± 1.4	17.7 ± 10.6	23.6 ± 9.8	26.3 ± 11.2	<0.001	<0.001	<0.001

*P*_1_: healthy controls versus single-vessel CAD groups; *P*_2_: healthy participants and dual-vessel lesion CAD subjects; *P*_3_: healthy volunteers and multivessel occluded CAD patients; TCHO: total cholesterol; TG: triglyceride; HDL-C: high-density lipoprotein cholesterol; LDL-C: low-density lipoprotein cholesterol.

## Data Availability

Data are available on request.
